# Dimethyl 2-(methyl­amino­methyl­ene)malonate

**DOI:** 10.1107/S1600536808014773

**Published:** 2008-05-21

**Authors:** Martin Gróf, Jozef Kožíšek, Viktor Milata, Anton Gatial

**Affiliations:** a Institute of Physical Chemistry and Chemical Physics, Faculty of Chemical and Food Technology, Slovak University of Technology, Radlinského 9, SK-812 37 Bratislava, Slovak Republic; b Institute of Organic Chemistry, Catalysis and Petrochemistry, Faculty of Chemical and Food Technology, Slovak University of Technology, Radlinského 9, Bratislava 81237, Slovak Republic

## Abstract

In the title compound, C_7_H_11_NO_4_, which is an example of a push–pull alkene, a network of N—H⋯O and C—H⋯O inter­actions helps to establish the crystal structure. The investigated crystal turned out to be a non-merohedral twin with a ratio of twin components of 0.442 (3):0.558 (3). Two pairs of independent mol­ecules (*Z*′ = 4) are linked by inter­molecular N—H⋯O hydrogen bonds, forming independent chains; the chains are connected *via* inter­molecular C—H⋯O contacts, building a three-dimensional network.

## Related literature

For related literature, see: Bouzard (1990 ▶); Cook (1969 ▶); Dyke (1973 ▶); Freeman (1981 ▶); Gróf *et al.* (2008 ▶); Kálmán & Argay (1998 ▶); Bolte (2004 ▶).
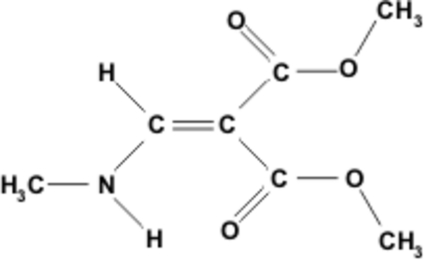


## Experimental

### 

#### Crystal data

C_7_H_11_NO_4_
*M* *_r_* = 173.17
Triclinic, 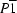

*a* = 11.165 (2) Å
*b* = 12.073 (2) Å
*c* = 13.211 (3) Å
α = 113.70 (3)°
β = 93.71 (3)°
γ = 94.02 (3)°
*V* = 1618.1 (6) Å^3^
*Z* = 8
Mo *K*α radiation
μ = 0.12 mm^−1^
*T* = 100 K
0.43 × 0.15 × 0.08 mm

#### Data collection

Oxford Diffraction GEMINI R diffractometer
Absorption correction: analytical (*CrysAlis RED*; Oxford Diffraction, 2006 ▶) *T*_min_ = 0.968, *T*_max_ = 0.996
9295 measured reflections
9295 independent reflections
3920 reflections with *I* > 2σ(*I*)

#### Refinement

*R*[*F* ^2^ > 2σ(*F* ^2^)] = 0.091
*wR*(*F* ^2^) = 0.257
*S* = 0.90
9295 reflections
446 parameters
96 restraints
H-atom parameters constrained
Δρ_max_ = 1.13 e Å^−3^
Δρ_min_ = −0.54 e Å^−3^

### 

Data collection: *CrysAlis CCD* (Oxford Diffraction, 2006 ▶); cell refinement: *CrysAlis CCD*; data reduction: *CrysAlis RED* (Oxford Diffraction, 2006 ▶); program(s) used to solve structure: *SHELXS97* (Sheldrick, 2008 ▶); program(s) used to refine structure: *SHELXL97* (Sheldrick, 2008 ▶); molecular graphics: *DIAMOND* (Brandenburg, 1998 ▶); software used to prepare material for publication: *enCIFer* (Allen *et al*., 2004 ▶).

## Supplementary Material

Crystal structure: contains datablocks global, I. DOI: 10.1107/S1600536808014773/si2088sup1.cif

Structure factors: contains datablocks I. DOI: 10.1107/S1600536808014773/si2088Isup2.hkl

Additional supplementary materials:  crystallographic information; 3D view; checkCIF report

## Figures and Tables

**Table 1:** Hydrogen-bond geometry (Å, °)

*D*—H⋯*A*	*D*—H	H⋯*A*	*D*⋯*A*	*D*—H⋯*A*
N1—H1⋯O1	0.86	2.08	2.689 (4)	127
N1—H1⋯O10	0.86	2.42	2.990 (4)	125
N2—H2⋯O5	0.86	2.08	2.687 (4)	127
N2—H2⋯O14^i^	0.86	2.30	2.893 (5)	127
N3—H3⋯O9	0.86	2.06	2.684 (4)	129
N3—H3⋯O2^i^	0.86	2.32	2.912 (5)	126
N4—H4⋯O13	0.86	2.08	2.698 (5)	128
N4—H4⋯O6	0.86	2.40	2.957 (5)	123
C4—H4*A*⋯O2	0.93	2.27	2.683 (7)	106
C6—H6*A*⋯O14	0.96	2.50	3.449 (7)	169
C11—H11*A*⋯O6	0.93	2.29	2.706 (6)	107
C11—H11*A*⋯O13	0.93	2.60	3.487 (6)	159
C14—H14*A*⋯O13	0.96	2.59	3.507 (6)	161
C18—H18*A*⋯O1	0.93	2.58	3.470 (6)	160
C18—H18*A*⋯O10	0.93	2.27	2.679 (6)	106
C25—H25*A*⋯O14	0.93	2.28	2.691 (6)	106
C27—H27*A*⋯O2^i^	0.96	2.59	3.340 (7)	135
C28—H28*C*⋯O6	0.96	2.60	3.024 (6)	107

Symmetry code: (i) 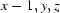.

**Fig. 1.**
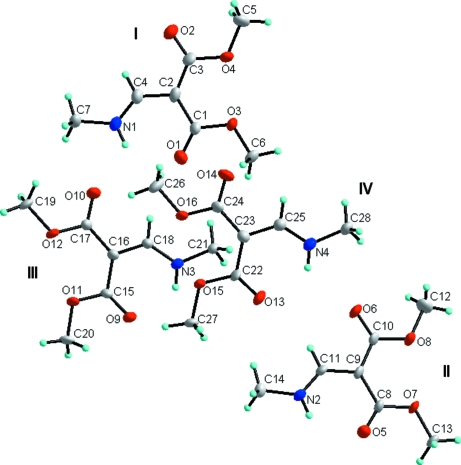
The atom-numbering scheme of dimethyl 2-(methylaminomethylene)malonate. Displacement ellipsoids are drawn at the 60% probability level.

**Fig. 2.**
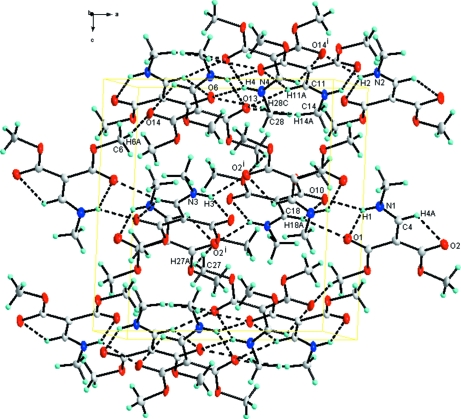
Packing diagram of dimethyl 2-(methylaminomethylene)malonate. Hydrogen-bond interactions are indicated by dashed lines.

## supplementary crystallographic information

### Comment

The title compound, C_7_ H_11_ N O_4_, belongs to the so-called push-pull olefins. Push-pull alkenes are substituted ethylenes containing electron-donor groups (D) at one end and electron-acceptor groups (A) at the other end of the general formula D^1^D^2^C=CA^1^A^2^. These compounds very often contain alkoxy, amino, alkylamino, dialkylamino or (hetero)aryl groups as electron-donor groups and cyano, acetyl, alkylester, methylsulfonyl or NO_2_ groups as electron-acceptor groups. They are useful as starting reactants or intermediates for a lot of pharmaceutical, polymer and other syntheses (Cook, 1969; Dyke, 1973). Mainly enamines are frequently used as reactants or intermediates in chemical syntheses of drugs, polymers and dyes (Bouzard, 1990). But also alkoxymethylenes are often used in organic synthesis (Freeman, 1981).

Chemical and physical properties of the title related structures were recently discussed (Gróf *et al.*,  2008 and literature cited therein).

The study of a similar compound, dimethyl 2-(aminomethylene)malonate, (Gróf *et al.*, 2008) revealed that this structure exists in the solid phase as EZ conformer (E denotes away from C=C double bond orientation of the carbonyl oxygen in *trans* position; *Z* denotes towards to C=C double bond orientation of the carbonyl oxygen in *cis* position). The title compound exists in the solid phase as ZZa conformer (a denotes anti orientation of the methylamino group, *e.g.* away from the C=C double bond orientation).

The molecules I and II, and molecules III and IV of the title compound (Fig.1) show pseudo translation (Kálmán & Argay, (1998).

### Experimental

To dimethyl 3-methoxymethylenemalonate (1.74 g, 10 mmol) in methanol (10 ml), an aqueous solution of methylamine (12 mmol) was added dropwise (amount according to concentration and density) over a period of 30 min with stirring. The slightly warmed mixture was stirred overnight at room temperature. The reaction mixture was then briefly heated to reflux (ca. 20 min). After ensuring that no starting derivative remained (thin-layer chromatography; Silufol 254, Kavalier Czechoslovakia; eluent chloroform-methanol 10:1 *v*/*v*, detection UV light 254 nm), the reaction mixture was evaporated on a vacuum evaporator and chromatographed on silica gel (eluent dichloromethane-methanol 10:1 *v*/*v*). Obtained product was recrystallized from minimal amount of chloroform and n-hexane mixture in refrigerator.

The solid phase mid-IR vibrational spectrum was recorded with a Nicolet model NEXUS 470 FTIR spectrometer at room temperature. The measurement was performed after mixing the powdered sample with KBr into a pellet.

The mid-IR vibrational frequencies of dimethyl 2-(methylaminomethylene)malonate are (in cm^-1^): 3301 m; 3199 w, sh, b; 3092 vw; 3052 vw; 3039 vw; 3013 w; 2999 w; 2951 m; 2924 w, sh; 2905 vw, sh; 1701 vw, sh; 1680 v s; 1651 vw, sh; 1631 v s; 1612 w, sh; 1541 vw; 1478 w; 1450 m; 1430 m; 1405 m; 1360 s; 1330 vw, sh; 1322 m; 1281 s; 1225 s; 1187 m; 1151 s; 1082 m; 1042 w; 1018 m; 998 w; 942 vw; 837 vw, sh; 820 s; 808 s; 768 m; 759 vw, sh; 671 m; 579 w, b; 459 vw; 446 vw; 413 m.

### Refinement

Olefinic and amino H atoms were positioned geometrically and allowed to ride on their corresponding parent atoms at distances of 0.93 and 0.86 Å, respectively, with *U*_iso_(H) = 1.2*U*_eq_(C,*N*). Methyl H atoms were located in a difference Fourier map and included in the model as a rigid rotating group, with C—H distance of 0.96 Å and with *U*_iso_(H) = 1.5*U*_eq_(C).

The investigated crystal was a non-merohedral twin. Two orientation matrices could be determined and the twin law was derived using the program TWINLAW (Bolte, 2004):

h(twin) = (1.00 * h) + (0.00 * k) + (0.00 * l)

k(twin) = (-0.15 * h) + (-1.00 * k) + (0.00 * l)

l(twin) = (-0.16 * h) + (0.00 * k) + (-1.00 *l).

For the refinement the reflection data file was modified using the program HKLF5 (Bolte, 2004). The contribution of the minor twin component refined to 0.442 (3).

### Crystal data

**Table:**

C_7_H_11_NO_4_	*Z* = 8
*M**_r_* = 173.17	*F*_000_ = 736
Triclinic, *P*1	*D*_x_ = 1.422 Mg m^−^^3^
Hall symbol: -P 1	Mo *K*α radiation λ = 0.71073 Å
*a* = 11.165 (2) Å	Cell parameters from 2135 reflections
*b* = 12.073 (2) Å	θ = 3.3–29.5º
*c* = 13.211 (3) Å	µ = 0.12 mm^−^^1^
α = 113.70 (3)º	*T* = 100 K
β = 93.71 (3)º	Block, yellow
γ = 94.02 (3)º	0.43 × 0.15 × 0.08 mm
*V* = 1618.1 (6) Å^3^	

### Data collection

**Table:**

Oxford Diffraction GEMINI R diffractometer	9295 independent reflections
Radiation source: fine-focus sealed tube	3920 reflections with *I* > 2σ(*I*)
Monochromator: graphite	*R*_int_ = 0.0000
*T* = 100 K	θ_max_ = 25.4º
Rotation method data acquisition using ω and φ scans	θ_min_ = 4.1º
Absorption correction: analytical(CrysAlis RED; Oxford Diffraction, 2006)	*h* = −13→13
*T*_min_ = 0.968, *T*_max_ = 0.996	*k* = −14→14
9295 measured reflections	*l* = −15→15

### Refinement

**Table:**

Refinement on *F*^2^	Secondary atom site location: difference Fourier map
Least-squares matrix: full	Hydrogen site location: inferred from neighbouring sites
*R*[*F*^2^ > 2σ(*F*^2^)] = 0.092	H-atom parameters constrained
*wR*(*F*^2^) = 0.257	*w* = 1/[σ^2^(*F*_o_^2^) + (0.1527*P*)^2^] where *P* = (*F*_o_^2^ + 2*F*_c_^2^)/3
*S* = 0.90	(Δ/σ)_max_ = 0.067
9295 reflections	Δρ_max_ = 1.13 e Å^−^^3^
446 parameters	Δρ_min_ = −0.54 e Å^−^^3^
96 restraints	Extinction correction: none
Primary atom site location: structure-invariant direct methods	

### Special details

**Table:**

Experimental. face-indexed (*CrysAlis RED*; Oxford Diffraction, 2006). 96 rigid bond restaints (DELU) were used in the refinement because the data to parameter ratio is low due to four independent molecules in the twinned structure.
Geometry. All e.s.d.'s (except the e.s.d. in the dihedral angle between two l.s. planes) are estimated using the full covariance matrix. The cell e.s.d.'s are taken into account individually in the estimation of e.s.d.'s in distances, angles and torsion angles; correlations between e.s.d.'s in cell parameters are only used when they are defined by crystal symmetry. An approximate (isotropic) treatment of cell e.s.d.'s is used for estimating e.s.d.'s involving l.s. planes.
Refinement. Refinement of *F*^2^ against ALL reflections. The weighted *R*-factor *wR* and goodness of fit *S* are based on *F*^2^, conventional *R*-factors *R* are based on *F*, with *F* set to zero for negative *F*^2^. The threshold expression of *F*^2^ > σ(*F*^2^) is used only for calculating *R*-factors(gt) *etc*. and is not relevant to the choice of reflections for refinement. *R*-factors based on *F*^2^ are statistically about twice as large as those based on *F*, and *R*- factors based on ALL data will be even larger.

### Fractional atomic coordinates and isotropic or equivalent isotropic displacement parameters (Å^2^)

**Table:**

	*x*	*y*	*z*	*U*_iso_*/*U*_eq_	
C1	1.0504 (4)	0.1221 (5)	0.6433 (4)	0.0202 (11)	
C2	1.1715 (5)	0.1334 (5)	0.6103 (4)	0.0234 (11)	
C3	1.2819 (5)	0.1043 (5)	0.6504 (4)	0.0238 (11)	
C4	1.1851 (5)	0.1766 (5)	0.5297 (4)	0.0274 (12)	
H4A	1.2634	0.1815	0.5106	0.033*	
C5	1.3825 (4)	0.0278 (5)	0.7690 (4)	0.0327 (14)	
H5C	1.3656	−0.0079	0.8203	0.039*	
H5B	1.4350	0.1019	0.8066	0.039*	
H5A	1.4210	−0.0279	0.7090	0.039*	
C6	0.9264 (4)	0.0767 (5)	0.7613 (4)	0.0241 (12)	
H6C	0.9311	0.0528	0.8224	0.029*	
H6B	0.8723	0.0182	0.7012	0.029*	
H6A	0.8970	0.1552	0.7848	0.029*	
C7	1.1353 (4)	0.2577 (5)	0.3932 (4)	0.0298 (13)	
H7C	1.1168	0.3404	0.4176	0.036*	
H7B	1.0889	0.2087	0.3236	0.036*	
H7A	1.2198	0.2546	0.3840	0.036*	
O1	0.9618 (3)	0.1470 (3)	0.6036 (3)	0.0317 (9)	
O2	1.3803 (3)	0.1235 (3)	0.6240 (3)	0.0294 (9)	
O3	1.0441 (3)	0.0828 (3)	0.7250 (3)	0.0257 (8)	
O4	1.2710 (3)	0.0540 (3)	0.7254 (3)	0.0303 (9)	
N1	1.1056 (4)	0.2113 (4)	0.4763 (3)	0.0290 (11)	
H1	1.0317	0.2069	0.4902	0.035*	
C8	0.0163 (4)	0.1936 (5)	1.0767 (4)	0.0227 (11)	
C9	0.1389 (4)	0.2097 (5)	1.0475 (4)	0.0198 (10)	
C10	0.2442 (4)	0.1583 (5)	1.0733 (4)	0.0234 (11)	
C11	0.1614 (4)	0.2782 (5)	0.9871 (4)	0.0224 (11)	
H11A	0.2405	0.2835	0.9699	0.027*	
C12	0.3325 (4)	0.0431 (6)	1.1587 (4)	0.0374 (14)	
H12C	0.3077	−0.0189	1.1835	0.045*	
H12B	0.3855	0.1061	1.2165	0.045*	
H12A	0.3742	0.0082	1.0936	0.045*	
C13	−0.1214 (4)	0.1026 (5)	1.1526 (4)	0.0261 (13)	
H13C	−0.1269	0.0428	1.1833	0.031*	
H13B	−0.1767	0.0763	1.0866	0.031*	
H13A	−0.1415	0.1788	1.2061	0.031*	
C14	0.1222 (4)	0.3979 (5)	0.8809 (4)	0.0231 (12)	
H14C	0.1054	0.4812	0.9146	0.028*	
H14B	0.0770	0.3583	0.8092	0.028*	
H14A	0.2068	0.3949	0.8728	0.028*	
O5	−0.0631 (3)	0.2510 (3)	1.0591 (3)	0.0302 (9)	
O6	0.3444 (3)	0.1722 (3)	1.0442 (3)	0.0350 (10)	
O7	−0.0002 (3)	0.1176 (3)	1.1253 (3)	0.0272 (9)	
O8	0.2281 (3)	0.0937 (3)	1.1328 (3)	0.0290 (9)	
N2	0.0879 (3)	0.3365 (4)	0.9506 (3)	0.0262 (10)	
H2	0.0153	0.3386	0.9690	0.031*	
C15	0.5457 (4)	0.3104 (5)	0.4300 (4)	0.0228 (11)	
C16	0.6700 (4)	0.2928 (5)	0.4565 (4)	0.0211 (11)	
C17	0.7792 (4)	0.3434 (5)	0.4301 (4)	0.0233 (11)	
C18	0.6930 (4)	0.2229 (5)	0.5142 (4)	0.0205 (11)	
H18A	0.7734	0.2125	0.5271	0.025*	
C19	0.8722 (4)	0.4640 (5)	0.3477 (4)	0.0289 (13)	
H19C	0.8530	0.5219	0.3178	0.035*	
H19B	0.9068	0.3981	0.2930	0.035*	
H19A	0.9291	0.5030	0.4126	0.035*	
C20	0.4082 (4)	0.4033 (5)	0.3540 (4)	0.0277 (13)	
H20C	0.4074	0.4651	0.3257	0.033*	
H20B	0.3676	0.4278	0.4203	0.033*	
H20A	0.3677	0.3284	0.2992	0.033*	
C21	0.6479 (4)	0.1022 (5)	0.6200 (4)	0.0203 (11)	
H21C	0.6040	0.0227	0.5890	0.024*	
H21B	0.6293	0.1455	0.6949	0.024*	
H21A	0.7329	0.0947	0.6197	0.024*	
O9	0.4592 (3)	0.2600 (3)	0.4495 (3)	0.0293 (9)	
O10	0.8808 (3)	0.3234 (3)	0.4529 (3)	0.0315 (9)	
O11	0.5322 (3)	0.3861 (3)	0.3795 (3)	0.0255 (8)	
O12	0.7640 (3)	0.4176 (3)	0.3774 (3)	0.0276 (9)	
N3	0.6133 (3)	0.1691 (4)	0.5531 (3)	0.0231 (10)	
H3	0.5379	0.1732	0.5389	0.028*	
C22	0.5158 (4)	0.3785 (5)	0.8567 (4)	0.0194 (10)	
C23	0.6390 (5)	0.3641 (5)	0.8899 (4)	0.0239 (11)	
C24	0.7487 (4)	0.3923 (5)	0.8491 (4)	0.0208 (11)	
C25	0.6589 (4)	0.3189 (5)	0.9702 (4)	0.0240 (11)	
H25A	0.7389	0.3117	0.9884	0.029*	
C26	0.8480 (4)	0.4731 (5)	0.7386 (4)	0.0313 (14)	
H26C	0.8301	0.5026	0.6823	0.038*	
H26B	0.8883	0.4010	0.7078	0.038*	
H26A	0.8992	0.5344	0.7993	0.038*	
C27	0.3829 (4)	0.4259 (5)	0.7369 (4)	0.0283 (13)	
H27C	0.3840	0.4511	0.6767	0.034*	
H27B	0.3486	0.4854	0.7977	0.034*	
H27A	0.3352	0.3488	0.7126	0.034*	
C28	0.6131 (4)	0.2394 (5)	1.1070 (4)	0.0288 (13)	
H28C	0.5723	0.1594	1.0857	0.035*	
H28B	0.5901	0.2928	1.1776	0.035*	
H28A	0.6988	0.2357	1.1127	0.035*	
O13	0.4292 (3)	0.3554 (3)	0.8977 (3)	0.0285 (9)	
O14	0.8494 (3)	0.3706 (3)	0.8760 (3)	0.0303 (9)	
O15	0.5064 (3)	0.4145 (3)	0.7733 (3)	0.0274 (9)	
O16	0.7371 (3)	0.4453 (3)	0.7781 (3)	0.0281 (9)	
N4	0.5799 (3)	0.2852 (4)	1.0234 (3)	0.0279 (11)	
H4	0.5048	0.2902	1.0087	0.033*	

### Atomic displacement parameters (Å^2^)

**Table:**

	*U*^11^	*U*^22^	*U*^33^	*U*^12^	*U*^13^	*U*^23^
C1	0.0197 (14)	0.022 (3)	0.023 (3)	−0.001 (2)	0.0012 (18)	0.015 (2)
C2	0.0238 (14)	0.027 (3)	0.028 (3)	0.004 (2)	0.010 (2)	0.018 (2)
C3	0.0194 (13)	0.024 (3)	0.032 (3)	−0.005 (2)	0.0010 (19)	0.016 (2)
C4	0.025 (2)	0.038 (4)	0.028 (3)	0.001 (2)	0.0052 (19)	0.021 (2)
C5	0.017 (2)	0.051 (4)	0.037 (3)	0.010 (3)	0.002 (2)	0.024 (3)
C6	0.016 (2)	0.029 (3)	0.033 (3)	0.003 (2)	0.010 (2)	0.017 (3)
C7	0.019 (3)	0.047 (4)	0.039 (3)	0.007 (3)	0.008 (2)	0.032 (3)
O1	0.0238 (14)	0.045 (3)	0.039 (2)	0.0054 (18)	0.0020 (16)	0.0304 (19)
O2	0.0227 (13)	0.035 (2)	0.037 (2)	0.0020 (17)	0.0098 (15)	0.0208 (19)
O3	0.0162 (16)	0.041 (2)	0.033 (2)	0.0036 (16)	0.0077 (14)	0.0271 (17)
O4	0.0117 (16)	0.050 (3)	0.043 (2)	0.0040 (16)	0.0044 (14)	0.0333 (18)
N1	0.022 (2)	0.040 (3)	0.037 (3)	0.000 (2)	0.0054 (17)	0.028 (2)
C8	0.0168 (15)	0.033 (3)	0.030 (3)	0.004 (2)	0.009 (2)	0.023 (2)
C9	0.0169 (15)	0.028 (3)	0.021 (3)	0.0014 (19)	0.009 (2)	0.015 (2)
C10	0.0171 (15)	0.039 (3)	0.024 (3)	0.004 (2)	0.009 (2)	0.022 (2)
C11	0.012 (2)	0.032 (3)	0.032 (3)	−0.0026 (19)	0.0027 (19)	0.022 (2)
C12	0.023 (3)	0.056 (4)	0.048 (4)	0.015 (3)	0.005 (3)	0.034 (3)
C13	0.014 (2)	0.037 (4)	0.038 (3)	0.002 (2)	0.010 (2)	0.026 (3)
C14	0.023 (3)	0.028 (3)	0.023 (3)	0.002 (2)	0.005 (2)	0.015 (2)
O5	0.0174 (15)	0.045 (3)	0.042 (2)	0.0075 (16)	0.0068 (16)	0.0311 (19)
O6	0.0161 (14)	0.058 (3)	0.050 (2)	0.0019 (18)	0.0111 (17)	0.041 (2)
O7	0.0144 (16)	0.043 (2)	0.040 (2)	0.0013 (15)	0.0094 (15)	0.0329 (18)
O8	0.0155 (17)	0.049 (3)	0.042 (2)	0.0103 (16)	0.0146 (15)	0.0355 (18)
N2	0.013 (2)	0.039 (3)	0.039 (3)	0.0046 (18)	0.0077 (18)	0.028 (2)
C15	0.0119 (11)	0.033 (3)	0.030 (3)	−0.0031 (19)	−0.005 (2)	0.022 (2)
C16	0.0128 (11)	0.032 (3)	0.027 (3)	0.0031 (19)	0.001 (2)	0.021 (2)
C17	0.0135 (12)	0.040 (3)	0.026 (3)	0.003 (2)	0.003 (2)	0.023 (2)
C18	0.012 (2)	0.031 (3)	0.025 (3)	0.0008 (19)	0.0000 (19)	0.019 (2)
C19	0.012 (2)	0.040 (4)	0.043 (3)	−0.004 (2)	0.005 (2)	0.026 (3)
C20	0.0096 (19)	0.041 (4)	0.042 (3)	0.003 (2)	−0.003 (2)	0.026 (3)
C21	0.013 (3)	0.029 (3)	0.025 (3)	−0.001 (2)	0.002 (2)	0.017 (2)
O9	0.0130 (12)	0.044 (2)	0.042 (2)	−0.0022 (16)	−0.0003 (16)	0.0305 (19)
O10	0.0138 (11)	0.051 (3)	0.044 (2)	0.0069 (16)	0.0047 (16)	0.033 (2)
O11	0.0089 (15)	0.041 (2)	0.040 (2)	0.0046 (14)	0.0015 (14)	0.0301 (18)
O12	0.0089 (16)	0.039 (2)	0.047 (2)	−0.0023 (14)	0.0013 (15)	0.0319 (18)
N3	0.013 (2)	0.030 (3)	0.036 (3)	0.0008 (17)	0.0019 (17)	0.022 (2)
C22	0.0149 (12)	0.021 (3)	0.028 (3)	−0.005 (2)	0.0005 (17)	0.017 (2)
C23	0.0187 (12)	0.033 (4)	0.026 (3)	0.002 (2)	0.0005 (19)	0.018 (2)
C24	0.0151 (13)	0.023 (3)	0.031 (3)	0.002 (2)	−0.0005 (19)	0.018 (2)
C25	0.017 (2)	0.032 (3)	0.030 (3)	0.002 (2)	0.0029 (18)	0.019 (2)
C26	0.020 (2)	0.042 (4)	0.033 (3)	−0.007 (3)	0.006 (2)	0.019 (3)
C27	0.0096 (19)	0.045 (4)	0.040 (3)	0.000 (2)	−0.001 (2)	0.028 (3)
C28	0.016 (3)	0.044 (4)	0.040 (3)	0.004 (2)	0.009 (2)	0.029 (3)
O13	0.0200 (13)	0.040 (2)	0.035 (2)	−0.0019 (17)	0.0086 (15)	0.0246 (18)
O14	0.0181 (12)	0.040 (3)	0.040 (2)	0.0072 (17)	−0.0039 (15)	0.0246 (19)
O15	0.0099 (15)	0.048 (3)	0.039 (2)	−0.0001 (16)	0.0013 (14)	0.0338 (19)
O16	0.0100 (16)	0.047 (3)	0.043 (2)	0.0014 (16)	0.0000 (14)	0.0347 (18)
N4	0.018 (2)	0.035 (3)	0.041 (3)	−0.0015 (19)	0.0029 (17)	0.026 (2)

### Geometric parameters (Å, °)

**Table:**

C1—O1	1.202 (5)	C15—O9	1.204 (5)
C1—O3	1.346 (5)	C15—O11	1.341 (6)
C1—C2	1.461 (6)	C15—C16	1.460 (6)
C2—C4	1.373 (6)	C16—C18	1.372 (6)
C2—C3	1.432 (7)	C16—C17	1.450 (7)
C3—O2	1.208 (5)	C17—O10	1.220 (5)
C3—O4	1.362 (6)	C17—O12	1.350 (6)
C4—N1	1.292 (6)	C18—N3	1.313 (6)
C4—H4A	0.9300	C18—H18A	0.9300
C5—O4	1.447 (5)	C19—O12	1.439 (5)
C5—H5C	0.9600	C19—H19C	0.9600
C5—H5B	0.9600	C19—H19B	0.9600
C5—H5A	0.9600	C19—H19A	0.9600
C6—O3	1.435 (5)	C20—O11	1.452 (5)
C6—H6C	0.9600	C20—H20C	0.9600
C6—H6B	0.9600	C20—H20B	0.9600
C6—H6A	0.9600	C20—H20A	0.9600
C7—N1	1.465 (6)	C21—N3	1.467 (6)
C7—H7C	0.9600	C21—H21C	0.9600
C7—H7B	0.9600	C21—H21B	0.9600
C7—H7A	0.9600	C21—H21A	0.9600
N1—H1	0.8600	N3—H3	0.8600
C8—O5	1.228 (5)	C22—O13	1.207 (5)
C8—O7	1.326 (6)	C22—O15	1.338 (5)
C8—C9	1.466 (6)	C22—C23	1.459 (6)
C9—C11	1.383 (6)	C23—C25	1.388 (7)
C9—C10	1.450 (6)	C23—C24	1.443 (7)
C10—O6	1.228 (5)	C24—O14	1.235 (5)
C10—O8	1.324 (5)	C24—O16	1.335 (6)
C11—N2	1.305 (6)	C25—N4	1.302 (6)
C11—H11A	0.9300	C25—H25A	0.9300
C12—O8	1.438 (5)	C26—O16	1.444 (5)
C12—H12C	0.9600	C26—H26C	0.9600
C12—H12B	0.9600	C26—H26B	0.9600
C12—H12A	0.9600	C26—H26A	0.9600
C13—O7	1.440 (5)	C27—O15	1.465 (5)
C13—H13C	0.9600	C27—H27C	0.9600
C13—H13B	0.9600	C27—H27B	0.9600
C13—H13A	0.9600	C27—H27A	0.9600
C14—N2	1.449 (6)	C28—N4	1.460 (6)
C14—H14C	0.9600	C28—H28C	0.9600
C14—H14B	0.9600	C28—H28B	0.9600
C14—H14A	0.9600	C28—H28A	0.9600
N2—H2	0.8600	N4—H4	0.8600
			
O1—C1—O3	121.1 (4)	O9—C15—O11	120.7 (4)
O1—C1—C2	124.2 (5)	O9—C15—C16	123.5 (5)
O3—C1—C2	114.7 (4)	O11—C15—C16	115.8 (4)
C4—C2—C3	113.4 (5)	C18—C16—C17	112.7 (4)
C4—C2—C1	117.9 (5)	C18—C16—C15	119.9 (4)
C3—C2—C1	128.7 (5)	C17—C16—C15	127.4 (5)
O2—C3—O4	120.0 (5)	O10—C17—O12	119.6 (4)
O2—C3—C2	124.8 (5)	O10—C17—C16	124.3 (5)
O4—C3—C2	115.2 (4)	O12—C17—C16	116.1 (4)
N1—C4—C2	129.8 (5)	N3—C18—C16	126.8 (4)
N1—C4—H4A	115.1	N3—C18—H18A	116.6
C2—C4—H4A	115.1	C16—C18—H18A	116.6
O4—C5—H5C	109.5	O12—C19—H19C	109.5
O4—C5—H5B	109.5	O12—C19—H19B	109.5
H5C—C5—H5B	109.5	H19C—C19—H19B	109.5
O4—C5—H5A	109.5	O12—C19—H19A	109.5
H5C—C5—H5A	109.5	H19C—C19—H19A	109.5
H5B—C5—H5A	109.5	H19B—C19—H19A	109.5
O3—C6—H6C	109.5	O11—C20—H20C	109.5
O3—C6—H6B	109.5	O11—C20—H20B	109.5
H6C—C6—H6B	109.5	H20C—C20—H20B	109.5
O3—C6—H6A	109.5	O11—C20—H20A	109.5
H6C—C6—H6A	109.5	H20C—C20—H20A	109.5
H6B—C6—H6A	109.5	H20B—C20—H20A	109.5
N1—C7—H7C	109.5	N3—C21—H21C	109.5
N1—C7—H7B	109.5	N3—C21—H21B	109.5
H7C—C7—H7B	109.5	H21C—C21—H21B	109.5
N1—C7—H7A	109.5	N3—C21—H21A	109.5
H7C—C7—H7A	109.5	H21C—C21—H21A	109.5
H7B—C7—H7A	109.5	H21B—C21—H21A	109.5
C1—O3—C6	115.1 (4)	C15—O11—C20	115.3 (4)
C3—O4—C5	115.6 (4)	C17—O12—C19	115.9 (4)
C4—N1—C7	123.2 (4)	C18—N3—C21	122.5 (4)
C4—N1—H1	118.4	C18—N3—H3	118.7
C7—N1—H1	118.4	C21—N3—H3	118.7
O5—C8—O7	123.5 (4)	O13—C22—O15	122.7 (4)
O5—C8—C9	120.8 (5)	O13—C22—C23	123.1 (5)
O7—C8—C9	115.7 (4)	O15—C22—C23	114.2 (4)
C11—C9—C10	113.6 (4)	C25—C23—C24	113.1 (5)
C11—C9—C8	119.4 (5)	C25—C23—C22	119.3 (5)
C10—C9—C8	127.0 (4)	C24—C23—C22	127.6 (5)
O6—C10—O8	119.7 (5)	O14—C24—O16	119.8 (4)
O6—C10—C9	124.2 (5)	O14—C24—C23	124.0 (5)
O8—C10—C9	116.1 (4)	O16—C24—C23	116.2 (4)
N2—C11—C9	129.5 (4)	N4—C25—C23	128.4 (5)
N2—C11—H11A	115.3	N4—C25—H25A	115.8
C9—C11—H11A	115.3	C23—C25—H25A	115.8
O8—C12—H12C	109.5	O16—C26—H26C	109.5
O8—C12—H12B	109.5	O16—C26—H26B	109.5
H12C—C12—H12B	109.5	H26C—C26—H26B	109.5
O8—C12—H12A	109.5	O16—C26—H26A	109.5
H12C—C12—H12A	109.5	H26C—C26—H26A	109.5
H12B—C12—H12A	109.5	H26B—C26—H26A	109.5
O7—C13—H13C	109.5	O15—C27—H27C	109.5
O7—C13—H13B	109.5	O15—C27—H27B	109.5
H13C—C13—H13B	109.5	H27C—C27—H27B	109.5
O7—C13—H13A	109.5	O15—C27—H27A	109.5
H13C—C13—H13A	109.5	H27C—C27—H27A	109.5
H13B—C13—H13A	109.5	H27B—C27—H27A	109.5
N2—C14—H14C	109.5	N4—C28—H28C	109.5
N2—C14—H14B	109.5	N4—C28—H28B	109.5
H14C—C14—H14B	109.5	H28C—C28—H28B	109.5
N2—C14—H14A	109.5	N4—C28—H28A	109.5
H14C—C14—H14A	109.5	H28C—C28—H28A	109.5
H14B—C14—H14A	109.5	H28B—C28—H28A	109.5
C8—O7—C13	114.7 (4)	C22—O15—C27	114.7 (4)
C10—O8—C12	116.1 (4)	C24—O16—C26	115.3 (4)
C11—N2—C14	123.4 (4)	C25—N4—C28	122.9 (4)
C11—N2—H2	118.3	C25—N4—H4	118.6
C14—N2—H2	118.3	C28—N4—H4	118.6
			
O1—C1—C2—C4	0.8 (8)	O9—C15—C16—C18	−5.1 (8)
O3—C1—C2—C4	−178.1 (5)	O11—C15—C16—C18	176.0 (5)
O1—C1—C2—C3	−178.7 (5)	O9—C15—C16—C17	175.9 (6)
O3—C1—C2—C3	2.4 (8)	O11—C15—C16—C17	−3.0 (7)
C4—C2—C3—O2	5.5 (8)	C18—C16—C17—O10	2.3 (8)
C1—C2—C3—O2	−175.0 (5)	C15—C16—C17—O10	−178.6 (5)
C4—C2—C3—O4	−176.0 (5)	C18—C16—C17—O12	−177.3 (4)
C1—C2—C3—O4	3.5 (8)	C15—C16—C17—O12	1.8 (8)
C3—C2—C4—N1	−179.8 (5)	C17—C16—C18—N3	177.3 (5)
C1—C2—C4—N1	0.6 (9)	C15—C16—C18—N3	−1.9 (8)
O1—C1—O3—C6	−1.6 (7)	O9—C15—O11—C20	1.4 (7)
C2—C1—O3—C6	177.3 (4)	C16—C15—O11—C20	−179.7 (4)
O2—C3—O4—C5	0.4 (7)	O10—C17—O12—C19	2.6 (7)
C2—C3—O4—C5	−178.1 (4)	C16—C17—O12—C19	−177.8 (4)
C2—C4—N1—C7	178.8 (5)	C16—C18—N3—C21	−176.1 (5)
O5—C8—C9—C11	−8.2 (7)	O13—C22—C23—C25	1.8 (8)
O7—C8—C9—C11	173.8 (5)	O15—C22—C23—C25	−175.5 (5)
O5—C8—C9—C10	173.2 (5)	O13—C22—C23—C24	−178.0 (5)
O7—C8—C9—C10	−4.7 (7)	O15—C22—C23—C24	4.8 (8)
C11—C9—C10—O6	−0.6 (8)	C25—C23—C24—O14	5.0 (8)
C8—C9—C10—O6	178.0 (5)	C22—C23—C24—O14	−175.2 (5)
C11—C9—C10—O8	178.8 (4)	C25—C23—C24—O16	−174.8 (4)
C8—C9—C10—O8	−2.6 (8)	C22—C23—C24—O16	4.9 (8)
C10—C9—C11—N2	−179.9 (5)	C24—C23—C25—N4	179.9 (5)
C8—C9—C11—N2	1.3 (8)	C22—C23—C25—N4	0.1 (9)
O5—C8—O7—C13	2.5 (7)	O13—C22—O15—C27	1.1 (7)
C9—C8—O7—C13	−179.6 (4)	C23—C22—O15—C27	178.3 (4)
O6—C10—O8—C12	−0.3 (7)	O14—C24—O16—C26	−0.1 (7)
C9—C10—O8—C12	−179.8 (4)	C23—C24—O16—C26	179.8 (4)
C9—C11—N2—C14	−175.4 (5)	C23—C25—N4—C28	−179.7 (5)

### Hydrogen-bond geometry (Å, °)

**Table:**

*D*—H···*A*	*D*—H	H···*A*	*D*···*A*	*D*—H···*A*
N1—H1···O1	0.86	2.08	2.689 (4)	127
N1—H1···O10	0.86	2.42	2.990 (4)	125
N2—H2···O5	0.86	2.08	2.687 (4)	127
N2—H2···O14^i^	0.86	2.30	2.893 (5)	127
N3—H3···O9	0.86	2.06	2.684 (4)	129
N3—H3···O2^i^	0.86	2.32	2.912 (5)	126
N4—H4···O13	0.86	2.08	2.698 (5)	128
N4—H4···O6	0.86	2.40	2.957 (5)	123
C4—H4A···O2	0.93	2.27	2.683 (7)	106
C6—H6A···O14	0.96	2.50	3.449 (7)	169
C11—H11A···O6	0.93	2.29	2.706 (6)	107
C11—H11A···O13	0.93	2.60	3.487 (6)	159
C14—H14A···O13	0.96	2.59	3.507 (6)	161
C18—H18A···O1	0.93	2.58	3.470 (6)	160
C18—H18A···O10	0.93	2.27	2.679 (6)	106
C25—H25A···O14	0.93	2.28	2.691 (6)	106
C27—H27A···O2^i^	0.96	2.59	3.340 (7)	135
C28—H28C···O6	0.96	2.60	3.024 (6)	107

Symmetry codes:  (i) *x*−1, *y*, *z*.
